# Síndrome de Kounis Tipo II após Exposição ao Diclofenaco: Desvendando a Ligação entre Alergia Medicamentosa e Síndrome Coronariana Aguda

**DOI:** 10.36660/abc.20250698

**Published:** 2026-04-14

**Authors:** Rafaella Avakian Mansur, Solange Desirée Avakian, Antonio de Padua Mansur

**Affiliations:** 1 Universidade de São Paulo São Paulo SP Brasil Universidade de São Paulo São Paulo, SP – Brasil; 2 Hospital das Clínicas Faculdade de Medicina Universidade de São Paulo São Paulo SP Brasil Instituto do Coração do Hospital das Clínicas da Faculdade de Medicina da Universidade de São Paulo, São Paulo, SP – Brasil

**Keywords:** Síndrome de Kounis, Diclofenaco, Hipersensibilidade a Drogas, Infarto do Miocárdio

## Introdução

A síndrome de Kounis (SK), identificada pela primeira vez em 1991 como “angina alérgica”, representa uma importante intersecção entre cardiologia e alergologia. Envolve episódios de síndrome coronariana aguda (SCA), como vasoespasmo, infarto do miocárdio ou trombose de stent, que ocorrem durante reações alérgicas, anafiláticas ou de hipersensibilidade.^1^ A SK demonstra o impacto direto da inflamação sistêmica nos vasos coronários, principalmente por meio da ativação e degranulação de mastócitos.^2^ Embora tenha sido descrita pela primeira vez há mais de 30 anos, a SK permanece altamente subdiagnosticada e frequentemente negligenciada, levando a atrasos no tratamento adequado.^3^ Sua fisiopatologia envolve a liberação de vários mediadores inflamatórios, como histamina, leucotrienos, triptase, quimase e fator de ativação de plaquetas. Essas substâncias podem causar vasoespasmo coronário, desencadear erosão ou ruptura de placas ateromatosas preexistentes e promover um ambiente protrombótico, resultando em SCA.^2 , 4^ Revisões sistemáticas da literatura existente, compostas principalmente por relatos e séries de casos, vêm esclarecendo as características epidemiológicas e clínicas da SK. Uma revisão abrangente recente de Cahuapaza-Gutierrez et al., incluindo 214 pacientes, mostrou predominância masculina (69,6%), idade média de 54,4 anos e hipertensão como comorbidade mais comum (33,6%).^3^ Os gatilhos mais frequentes são medicamentos (38,3%), especialmente antibióticos e anti-inflamatórios não esteroides, seguidos por picadas de insetos (26,2%).^4^ A SK é classificada em três tipos principais: tipo I (espasmo coronário sem doença preexistente), tipo II (infarto do miocárdio na presença de doença arterial coronariana subjacente) e tipo III (trombose de stent).^2^ Um “tipo IV” proposto envolve trombose de enxertos de revascularização do miocárdio. Reconhecer essa classificação é crucial para os médicos, pois o tratamento deve abordar tanto a isquemia cardíaca aguda quanto a reação alérgica. O presente relato de caso de SK tipo II típica desencadeada por diclofenaco sódico visa contribuir para o crescente corpo de evidências e ressaltar a importância de maior conscientização clínica.

### Apresentação do caso

Uma mulher na faixa dos 50 anos compareceu ao pronto-socorro após uma reação adversa aguda, aproximadamente 15 minutos após ingerir um único comprimido de diclofenaco sódico para controle da dor. Seus sintomas iniciais incluíram erupção cutânea generalizada, hipotensão profunda e síncope.

### Apresentação inicial e manejo agudo

**Figura 1:**
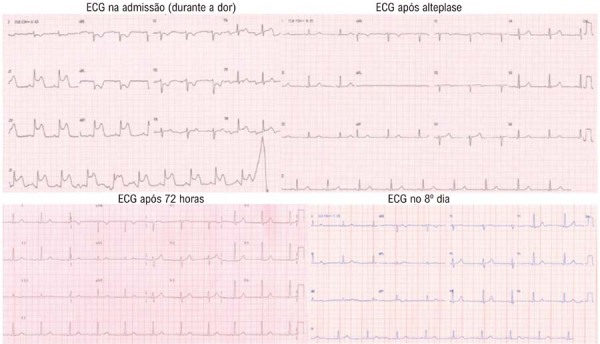
– Eletrocardiogramas demonstrando elevação do segmento ST nas derivações inferiores (II, III e aVF) na admissão e no 8º dia de internação.

**Figura 2:**
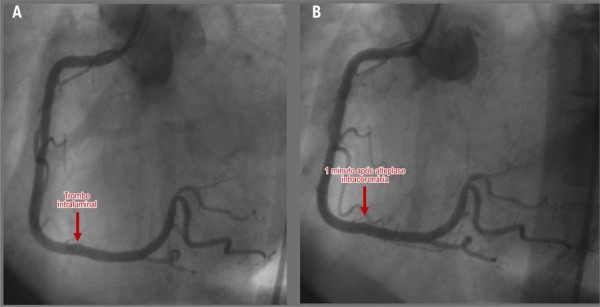
– Angiografia coronária mostrando trombo na artéria coronária direita (A) e sua resolução após trombólise intracoronária (B).

Ao chegar, a paciente apresentava sudorese e encontrava-se em mau estado geral. Seus sinais vitais indicavam hipotensão (pressão arterial: 80/50 mmHg) e taquicardia (frequência cardíaca: 110 bpm). O exame físico revelou erupção urticariforme difusa. Foi realizado imediatamente um eletrocardiograma de 12 derivações, demonstrando elevação significativa do segmento ST nas derivações inferiores (II, III e aVF) ( Figura 1 ). Foi estabelecido o diagnóstico de infarto agudo do miocárdio com supradesnivelamento do segmento ST nas derivações inferiores. A paciente foi transferida com urgência para o laboratório de cateterismo cardíaco. A angiografia coronária de emergência mostrou função ventricular esquerda normal e identificou um trombo discreto no segmento médio-distal da artéria coronária direita (ACD), sem evidência de aterosclerose significativa, definida como lesões coronárias com estenose luminal superior a 70% ou ruptura de placa naquele momento ( Figura 2A ). A trombólise intracoronária com alteplase foi administrada com sucesso. Uma angiografia de acompanhamento confirmou a repermeabilização completa da ACD, sem trombo residual ou lesões que limitassem o fluxo ( Figura 2B ).

### Histórico médico antecedente e evolução hospitalar

Seu histórico médico relevante incluía hipertensão arterial sistêmica e tabagismo de 20 cigarros por dia, que havia abandonado antes do evento. Ela não tinha alergias medicamentosas conhecidas. Após o procedimento, foi estabilizada na unidade coronariana. Iniciou-se um regime de prevenção secundária com atenolol, sinvastatina e aspirina (AAS). Após uma semana de internação sem intercorrências, recebeu alta assintomática, com pressão arterial bem controlada (120/80 mmHg) e frequência cardíaca de 56 bpm. Seu índice de massa corporal (IMC) era de 28,31 kg/m^2^ e o exame físico na alta foi normal.

### Acompanhamento em curto e longo prazo

Após 2 meses, foram realizados exames laboratoriais abrangentes para identificar condições protrombóticas subjacentes. A triagem para trombofilia foi negativa para fator V de Leiden, proteína C, proteína S, anticoagulante lúpico e anticorpos antinucleares. Seu perfil lipídico mostrou triglicerídeos de 118 mg/dL, colesterol total de 189 mg/dL, HDL de 72 mg/dL, LDL de 93 mg/dL e lipoproteína(a) de 13,9 mg/dL. Seu nível de homocisteína estava dentro da faixa normal, em 11,8 μmol/L. Após 15 anos de acompanhamento, ela apresentou um episódio típico de angina. Um ecocardiograma evidenciou fração de ejeção do ventrículo esquerdo preservada em 61%, pressão sistólica da artéria pulmonar de 32 mmHg e disfunção diastólica de grau I. O ultrassom Doppler de carótidas identificou alterações ateromatosas sem estenose significativa. A angiotomografia computadorizada coronária (angioTC) mostrou aterosclerose avançada com escore de cálcio de Agatston de 467 e calcificações difusas nas artérias coronárias, juntamente com estenose moderada (50% a 70%) no ramo ventricular posterior da ACD. Dois anos depois, ela foi diagnosticada com doença vascular periférica causada por aterosclerose nos membros inferiores, incluindo oclusão do segmento médio da artéria tibial posterior esquerda e desenvolvimento de circulação colateral. Seu IMC havia aumentado para 32,67 kg/m^2^.

### Quadro final

Em sua avaliação mais recente, a paciente permanecia livre de sintomas cardiovasculares. Seu regime medicamentoso de longo prazo foi otimizado e atualmente inclui AAS 100 mg ao dia, rosuvastatina 20 mg ao dia e verapamil 160 mg ao dia para manejo contínuo do risco cardiovascular.

## Discussão

O caso relatado envolve uma mulher na faixa dos 50 anos que desenvolveu um infarto agudo do miocárdio com supradesnivelamento do segmento ST secundário a uma reação de hipersensibilidade induzida por diclofenaco, exemplificando a SK tipo II. A apresentação da paciente está em consonância com a literatura: início rápido de sintomas alérgicos (erupção cutânea e hipotensão) seguido de isquemia cardíaca, com evidência angiográfica de formação de trombo em contexto de doença arterial coronariana pré-existente, porém não obstrutiva, posteriormente confirmada por tomografia coronariana.^4 - 6^ O caso relatado oferece uma oportunidade para examinar aspectos fundamentais da SK. Em primeiro lugar, o gatilho — o diclofenaco — é um AINE reconhecidamente implicado na SK. Uma revisão sistemática de Pejcic et al., focada especificamente na SK relacionada ao diclofenaco, identificou 20 casos, sendo a elevação do segmento ST o achado eletrocardiográfico mais comum (85%) e a hipotensão observada em 75% dos pacientes.^7^ Embora a revisão tenha relatado uma predominância masculina de 90%, nosso caso demonstra que a SK pode afetar ambos os sexos, e um alto grau de suspeita deve ser mantido independentemente do sexo. O mecanismo provável envolve duas vias principais. Como um AINE, o diclofenaco pode provocar reações de hipersensibilidade por meio de mecanismos mediados por IgE ou, mais comumente, por meio da inibição farmacológica da COX-1, que direciona o metabolismo do ácido araquidônico para a via da lipoxigenase, aumentando a produção de leucotrienos cisteinílicos e outros mediadores inflamatórios.^8 , 9^ Esses mediadores, liberados pelos mastócitos, causam vasoconstrição intensa (histamina e leucotrienos), aumentam a permeabilidade vascular, levando à hipotensão, e ativam as plaquetas.^4 , 10^ Em vasos com disfunção endotelial preexistente ou aterosclerose subclínica, essa resposta inflamatória pode desencadear erosão da placa e formação aguda de trombo, como observado em nossa paciente. A ausência de estenose significativa após a trombólise reforça a ideia de que o processo é dinâmico, impulsionado por vasoespasmo e trombose, e não por uma lesão obstrutiva fixa. Do ponto de vista do tratamento, o caso relatado destaca o sucesso da terapia de dupla via, abordando simultaneamente a resposta alérgica e o evento coronariano. O uso da trombólise intracoronária foi crucial para a resolução do trombo. É importante ressaltar o debate em curso sobre o uso de adrenalina na SK, visto que ela pode agravar o vasoespasmo coronariano por meio da estimulação dos receptores α-adrenérgicos.^4^ Isso enfatiza a necessidade de um manejo individualizado e do desenvolvimento de diretrizes clínicas formais. Além disso, o desfecho a longo prazo da paciente foi favorável, em consonância com dados de revisões sistemáticas que indicam que a maioria dos pacientes com SK (86,92%) apresenta bom prognóstico com tratamento imediato.^3^ O caso relatado ressalta que a SK não é tão rara quanto se pensa, mas é frequentemente negligenciada. Maior conscientização da comunidade médica e a consideração sistemática da SK em qualquer paciente que apresente sintomas de SCA, juntamente com características alérgicas, são cruciais para melhorar o diagnóstico e o manejo.

### Prognóstico a longo prazo e identificação da doença subjacente

O acompanhamento a longo prazo da nossa paciente ao longo de 15 anos proporciona uma perspectiva rara e valiosa sobre os efeitos duradouros da SK. Inicialmente tratada como um evento agudo e reversível, a evolução clínica subsequente da paciente revelou uma carga aterosclerótica extensa e progressiva. Isso foi claramente documentado pela angioTC, que mostrou um escore de cálcio de Agatston de 467, e estenose moderada, confirmando doença arterial coronariana subjacente significativa, não aparente durante o episódio trombótico inicial. Essa progressão destaca um aspecto fundamental da SK tipo II: ela frequentemente revela um substrato coronariano vulnerável e silencioso, propenso a futuros eventos cardiovasculares.^2^ A relevância desse achado a longo prazo reside na compreensão de que os pacientes que sobrevivem a um episódio de SK não são “curados” após a resolução das fases alérgica e trombótica agudas. Em vez disso, eles apresentam risco substancial de progressão aterosclerótica típica. O ambiente inflamatório sistêmico durante o evento anafilático, caracterizado por mediadores derivados de mastócitos, como triptase e quimase, pode não apenas desencadear o evento coronariano agudo, mas também contribuir para a desestabilização e o crescimento a longo prazo das placas ateroscleróticas.^9 , 10^ Além disso, o desenvolvimento de doença arterial periférica sintomática em nossa paciente destaca que a vulnerabilidade é sistêmica, afetando todo o sistema vascular. Portanto, o manejo da SK deve ir além da fase aguda. Como ilustrado neste caso, a prevenção secundária agressiva e ao longo da vida é essencial. Isso inclui o controle rigoroso dos fatores de risco tradicionais e a adesão consistente à terapia antiplaquetária e às estatinas de alta intensidade, conforme recomendado pelas diretrizes padrão para doença arterial coronariana.^11^ A manutenção do estado assintomático a longo prazo de nossa paciente provavelmente resulta dessa abordagem farmacológica contínua. A SK deve, portanto, ser vista não apenas como uma crise alérgica e cardíaca aguda, mas como um sinal de alerta significativo de doença cardiovascular subjacente e potencialmente progressiva, exigindo monitoramento contínuo e manejo dos fatores de risco ao longo da vida.

A extensa doença aterosclerótica da paciente, 15 anos após o evento inicial, não pode ser atribuída exclusivamente ao episódio anterior de SK. Ela apresentava diversos fatores de risco cardiovascular: tabagismo prévio (com exposição incerta durante o acompanhamento), controle inadequado do colesterol LDL no primeiro mês de acompanhamento e possível desenvolvimento de hipertensão, diabetes, sobrepeso e outros riscos comportamentais, socioeconômicos ou ambientais. Além disso, era provável que estivesse na pós-menopausa, o que aumentava ainda mais seu risco cardiovascular. Portanto, atribuir sua carga aterosclerótica posterior inteiramente ao episódio de SK seria inapropriado. Ademais, a sugestão de uma ligação entre a atividade dos mastócitos e a aterosclerose envolve exposição crônica, o que é improvável na ausência de sintomas alérgicos recorrentes durante o acompanhamento. Embora a SK esteja sendo relatada com mais frequência, ainda faltam estudos prospectivos e diretrizes específicas para estabelecê-la como um indicador definitivo de vulnerabilidade aterosclerótica. Apesar de o episódio subsequente ao uso de diclofenaco poder sugerir tal conexão, especialmente em pacientes com doença arterial coronariana preexistente, essa associação não alcançou consenso clínico e não é sustentada por evidências robustas.

## Conclusão

Os médicos devem considerar a SK em pacientes com sintomas coronarianos agudos após exposição a alérgenos conhecidos, incluindo medicamentos comuns como o diclofenaco. A identificação precoce e o tratamento combinado dos componentes alérgico e cardíaco são cruciais para prevenir complicações e melhorar os desfechos.
