# Supplementary material for: Síndrome de Kounis Tipo II após Exposição ao Diclofenaco: Desvendando a Ligação entre Alergia Medicamentosa e Síndrome Coronariana Aguda
Source: Arq Bras Cardiol. 2026 Apr 14;123(3):e20250698. [Article in Portuguese] doi: 10.36660/abc.20250698 (PMC13128209; doi:10.36660/abc.20250698)
Supplement: Translation

# Translation: Kounis Syndrome Type II after Diclofenac Exposure: Unmasking a Link Between Drug Allergy and Acute Coronary Syndrome

**DOI:** 10.36660/abc.20250698i

**Authors:** Rafaella Avakian Mansur, Solange Desirée Avakian, Antonio de Padua Mansur

**Affiliations:** 1 Brazil Universidade de São Paulo São Paulo, SP – Brazil; 2 Brazil Instituto do Coração do Hospital das Clínicas da Faculdade de Medicina da Universidade de São Paulo, São Paulo, SP – Brazil

**Keywords:** Kounis Syndrome, Diclofenac, Drug Hypersensitivity, Myocardial Infarction

## Introduction

Kounis syndrome (KS), first identified in 1991 as “allergic angina,” marks a significant intersection between cardiology and allergology. It involves episodes of acute coronary syndrome (ACS), such as vasospasm, myocardial infarction, or stent thrombosis, occurring during allergic, anaphylactic, or hypersensitivity reactions.^1^This syndrome demonstrates the direct impact of systemic inflammation on coronary vessels, primarily through mast cell activation and degranulation.^2^Although it was first described more than 30 years ago, KS remains highly underdiagnosed and frequently overlooked, leading to delays in proper treatment.^3^Its pathophysiology includes the release of various inflammatory mediators like histamine, leukotrienes, tryptase, chymase, and platelet-activating factor. These substances can cause coronary artery vasospasm, trigger plaque erosion or rupture in existing atheromatous plaques, and promote a prothrombotic environment, resulting in ACS.^2 , 4^Systematic reviews of existing literature, mainly case reports and series, are beginning to clarify KS’s epidemiological and clinical features. A recent comprehensive review by Cahuapaza-Gutierrez et al. of 214 patients showed a male predominance (69.6%), with an average age of 54.4 years, and hypertension was the most common comorbidity (33.6%).^3^The most frequent triggers are medications (38.3%), especially antibiotics and non-steroidal anti-inflammatory drugs (NSAIDs), followed by insect stings (26.2%).^4^KS is classified into three main types: type I (coronary spasm without pre-existing disease), type II (myocardial infarction with underlying coronary artery disease), and type III (stent thrombosis).^2^A proposed “type IV” involves thrombosis of coronary artery bypass grafts. Recognizing this classification is crucial for clinicians, as treatment must address both the acute cardiac ischemia and the allergic reaction. This case report of typical type II KS triggered by diclofenac sodium aims to contribute to the growing body of evidence and underscore the importance of heightened clinical awareness.

### Case presentation

A woman in her fifties presented to the emergency department, following an acute adverse reaction approximately 15 minutes after ingesting a single tablet of diclofenac sodium for pain management. Her initial symptoms included a generalized cutaneous eruption, profound hypotension, and syncope.

### Initial presentation and acute management

Upon arrival, the patient was diaphoretic and in distress. Her vital signs indicated hypotension (blood pressure: 80/50 mmHg) and tachycardia (heart rate: 110 bpm). Physical examination confirmed a diffuse urticarial rash. An immediate 12-lead electrocardiogram (ECG) showed significant ST-segment elevation in the inferior leads (II, III, and aVF) ( Figure 1 ). A diagnosis of acute inferior ST-elevation myocardial infarction was made. The patient was urgently transferred to the cardiac catheterization laboratory. Emergency coronary angiography showed normal left ventricular function and identified a discrete thrombus in the mid-to-distal segment of the right coronary artery (RCA), with no evidence of significant atherosclerosis, defined as coronary lesions greater than 70% luminal narrowing or plaque rupture at that time ( Figure 2A ). Intracoronary thrombolysis with alteplase was successfully administered. A follow-up angiogram confirmed complete reperfusion of the RCA with no residual thrombus or flow-limiting lesions ( Figure 2B ).

**Figure 1:** – Electrocardiograms showing ST-segment elevation in inferior leads (II, III, and aVF) at hospital admission and on day 8.

**Figure 2:** – Coronary angiography showing thrombus in the right coronary artery (A) and resolution after thrombolysis (B).

### Past medical history and in-hospital course

Her relevant medical history included systemic hypertension and a history of smoking 20 cigarettes daily, which she had quit prior to this event. She had no known drug allergies. After the procedure, she was stabilized in the coronary care unit. She was started on a secondary prevention regimen consisting of atenolol, simvastatin, and aspirin (ASA). Following a smooth 1-week hospital stay, she was discharged without symptoms, with well-controlled blood pressure (120/80 mmHg) and a heart rate of 56 bpm. Her body mass index (BMI) was 28.31 kg/m^2^, and physical examination at discharge was normal.

### Short- and long-term follow-up

After 2 months, extensive laboratory tests were conducted to identify underlying prothrombotic conditions. Thrombophilia screening was negative for factor V Leiden, protein C, protein S, lupus anticoagulant, and antinuclear antibodies. Her lipid profile showed triglycerides at 118 mg/dL, total cholesterol at 189 mg/dL, HDL at 72 mg/dL, LDL at 93 mg/dL, and lipoprotein(a) at 13.9 mg/dL. Her homocysteine level was within the normal range at 11.8 μmol/L. After 15 years of follow-up, she experienced a typical angina episode. An echocardiogram revealed a preserved left ventricular ejection fraction of 61%, pulmonary artery systolic pressure of 32 mmHg, and grade I diastolic dysfunction. Carotid Doppler ultrasound identified atheromatous changes without significant stenosis. Coronary computed tomography angiography (CCTA) showed advanced atherosclerosis with an Agatston calcium score of 467 and diffuse calcifications in the coronary arteries, along with a moderate (50% to 70%) stenosis in the posterior ventricular branch of the RCA. Two years later, she was diagnosed with peripheral vascular insufficiency caused by lower-limb atherosclerosis, including occlusion of the mid-segment of the left posterior tibial artery and development of collateral circulation. Her BMI had increased to 32.67 kg/m^2^.

### Final status

At her most recent evaluation, the patient remained free of cardiovascular symptoms. Her long-term medication regimen has been optimized and currently includes ASA 100 mg daily, rosuvastatin 20 mg daily, and verapamil 160 mg daily for ongoing cardiovascular risk management.

## Discussion

This case involves a woman in her fifties who developed an ST-elevation myocardial infarction secondary to a diclofenac-induced hypersensitivity reaction, exemplifying type II KS. The patient’s presentation aligns with the literature: rapid onset of allergic symptoms (rash and hypotension) followed by cardiac ischemia, with angiographic evidence of thrombus formation on a background of pre-existing but non-obstructive coronary artery disease, later confirmed by coronary tomography.^4 - 6^This case provides an opportunity to examine key aspects of KS. First, the trigger, diclofenac, is a well-known NSAID culprit. A systematic review by Pejcic et al. focusing specifically on diclofenac-related KS, identified 20 cases; ST-segment elevation was the most common ECG finding (85%), and hypotension was identified in 75% of patients.^7^Although the review reported a 90% male predominance, our case shows that KS can affect either sex, and high suspicion should be maintained regardless of sex. The likely mechanism involves a dual pathway. As an NSAID, diclofenac can provoke hypersensitivity reactions via IgE-mediated mechanisms or, more commonly, through pharmacologic COX-1 inhibition, which shifts arachidonic acid metabolism toward the lipoxygenase pathway, increasing the production of cysteinyl leukotrienes and other inflammatory mediators.^8 , 9^These mediators, released from mast cells, cause intense vasoconstriction (histamine and leukotrienes), increase vascular permeability leading to hypotension, and activate platelets.^4 , 10^In vessels with pre-existing endothelial dysfunction or subclinical atherosclerosis, this inflammatory response can trigger plaque erosion and acute thrombus formation, as seen in our patient. The lack of significant stenosis after thrombolysis supports the concept that the process is dynamic, driven by vasospasm and thrombosis rather than a fixed obstructive lesion. From a treatment perspective, this case highlights the successful use of dual-pathway therapy, addressing both the allergic response and the coronary event simultaneously. The use of intracoronary thrombolysis was critical to resolving the thrombus. It is important to note the ongoing debate about adrenaline use in KS, as it may worsen coronary vasospasm through α-adrenergic receptor stimulation.^4^This emphasizes the need for tailored management and the development of formal clinical guidelines. Additionally, the patient’s long-term outcome was favorable, consistent with systematic review data indicating most patients with KS (86.92%) have a good prognosis with prompt treatment.^3^This case underlines that KS is not as rare as often thought but is frequently overlooked. Increased clinician awareness and systematic consideration of KS in any patient presenting with ACS symptoms, along with allergic features, are crucial to improving diagnosis and management.

### Long-term prognosis and the identification of underlying disease

The long-term follow-up of our patient over 15 years provides rare and valuable insight into the lasting effects of KS. Initially treated as an acute and reversible event, the patient’s subsequent clinical course revealed a progressive and extensive atherosclerotic burden. This was clearly documented by CCTA, which showed an Agatston calcium score of 467, and moderate stenosis, confirming significant underlying coronary artery disease that was not apparent during the initial thrombotic episode. This progression highlights a key aspect of type II KS: it often uncovers a vulnerable, silent coronary substrate prone to future cardiovascular events.^2^The long-term importance lies in understanding that patients who survive an episode of KS are not “cured” once the acute allergic and thrombotic phases resolve. Instead, they carry a substantial risk for typical atherosclerotic progression. The systemic inflammatory environment during the anaphylactic event, characterized by mast cell-derived mediators such as tryptase and chymase, may not only trigger the acute coronary event but also contribute to long-term destabilization and growth of atherosclerotic plaques.^9 , 10^Additionally, our patient’s development of symptomatic peripheral artery disease highlights that vulnerability is systemic, affecting the entire vascular system. Therefore, management of KS must go beyond the acute phase. As illustrated in this case, aggressive and lifelong secondary prevention is essential. This includes strict control of traditional risk factors and consistent adherence to antiplatelet therapy and high-intensity statins, as recommended by standard coronary artery disease guidelines.^11^Our patient’s positive long-term symptomatic status likely results from this ongoing pharmacological approach. KS should, therefore, be viewed not just as an acute allergic and cardiac crisis but as a significant warning sign of underlying and potentially worsening cardiovascular disease, requiring continuous monitoring and management of risk factors throughout life.

The patient’s extensive atherosclerotic disease 15 years after the initial event cannot be solely attributed to the previous KS episode. She had exhibited several potential cardiovascular risk factors: former smoking (with uncertain exposure during follow-up), inadequate LDL-cholesterol management at 1 month, and possible development of hypertension, diabetes, overweight, and other behavioral, socioeconomic, or environmental risks. Additionally, she was likely postmenopausal, which further increased her cardiovascular risk. Therefore, attributing her later atherosclerotic burden entirely to the KS episode would be inappropriate. Furthermore, the suggested link between mast cell activity and atherosclerosis involves chronic exposure, which is unlikely in the absence of recurrent allergic symptoms during follow-up. Even though KS is being reported more often, it still lacks prospective studies and specific guidelines to establish it as a definitive indicator of atherosclerotic vulnerability. Although the episode following diclofenac use might suggest such a connection, especially in patients with existing coronary artery disease, this association has not achieved clinical consensus and is not strongly supported by solid evidence.

## Conclusion

Clinicians should consider KS in patients with acute coronary symptoms after exposure to known allergens, including common drugs such as diclofenac. Early identification and combined treatment of allergic and cardiac components are crucial to prevent complications and improve outcomes.
